# Advance Care Planning: A Retrospective Audit in a National Referral Center for Interstitial Lung Diseases

**DOI:** 10.1177/10499091241267914

**Published:** 2024-10-14

**Authors:** Lian Trapman, Marieke Zwakman, Everlien de Graaf, Lea M. Dijksman, Jan C. Grutters, Saskia C.C.M. Teunissen

**Affiliations:** 1 ILD Center of Excellence, Department of Pulmonology, 6028 St Antonius Hospital, Nieuwegein, Netherlands; 2 8119 University of Applied Sciences Utrecht, Utrecht, The Netherlands; 3 Center of Expertise Palliative Care Utrecht, Julius Center for Healthcare Sciences and Primary Care, Dept. General Practice, University Medical Center Utrecht, Utrecht, Netherlands; 4 Department of Value-Based Healthcare, St Antonius Hospital, Nieuwegein, The Netherlands; 5 Division of Heart and Lungs, 8124 University Medical Center Utrecht, Utrecht, Netherlands

**Keywords:** palliative care, advance care planning, interstitial lung diseases, pulmonary fibrosis, chart review

## Abstract

**Background:**

Idiopathic and progressive pulmonary fibrosis (IPF/PPF) of known cause are relatively rare lung diseases with a limited survival time after diagnosis. Conscious attention for palliative care is recommended. Optimal care requires collaboration to define goals and preferences for future medical treatment and care with the patient and their families, to inform (or enable) Advance Care Planning (ACP).

**Objective:**

To get insight into the frequency of key elements of ACP described after dialogues with patients with IPF/PPF.

**Methods:**

A retrospective audit included charts of patients with IPF/PPF who died between December 2017 and December 2020. A data extraction model was developed based on a guideline for patient federation and wider literature and finally consisted of fourteen key elements. Subsequently content analysis was performed.

**Results:**

The medical charts of 60 patients showed that an element of ACP was recorded in 57(95%) of cases. No medical chart contained all fourteen key elements of ACP. Most frequently recorded ACP elements were: knowledge of illness, goals of treatment and care and fears and concerns.

**Conclusion:**

The lack of structural implementation of ACP in the care for patients with interstitial lung disease, results in only some elements of ACP being dialogued by health care professionals (HCP). These notes recorded are often superficial and reflect the view of the HCP. Implementation of ACP conversations and structured documentation is needed to gain better insight into the wishes and preferences of the patient.

## Introduction

Idiopathic pulmonary fibrosis (IPF) and other forms of progressive pulmonary fibrosis (PPF) are relatively rare progressive fibrosing lung diseases,^1,2^ with an estimated prevalence and incidence of 0.33-4.51 and 0.09-1.30/10.000 persons.^3^ PPF can have many different causes such as autoimmune diseases and inhalation of antigens from the external environment. In the majority of patients, the disease cause remains elusive (IPF). The survival time after diagnosis IPF is median 4.5 years.^4,5^ The survival time of patients with a known cause differs widely. This depends on the effect of elimination of environmental trigger/antigen(s) such as in fibrosing hypersensitivity pneumonitis (fHP) and the tolerability and effect of immunosuppressive treatment in autoimmune-related PPF. More decline in Forced Vital Capacity (pulmonary function test, FVC) gives a shorter survival time, and can be even as poor as IPF.^6,7^

In case of IPF or if pulmonary fibrosis (PF) progresses the mainstay, therapeutic option is antifibrotic therapy, which can slow down disease progression but commonly can have many side effects, eg, diarrhea and the possibility of burning skin due to sunshine exposure.

For patients with an advanced stage of disease and a reduced quality of life, communication about goals and preferences for their remaining life is important to provide appropriate care. Advance Care Planning (ACP) is a communication process enabling individuals to define goals and preferences for future medical treatment and care and to discuss, record, and review these with family and caregivers.^8,9^

Elements which could be dialogued in ACP conversations are for example hopes, fears, goals of future medical treatment and care, religious wishes, and preferred final place of care and death.^8,10^

Due to the fact that mechanical ventilation in patients with IPF has a high change on progressing or exacerbating the disease and a high mortality risk,^11^ decisions about mechanical ventilation and cardio-pulmonary resuscitation is important.^10^

Several studies demonstrated a positive impact of ACP on quality of life, quality of death and dying in patients with life-limiting diseases^12,13^ Although there have not been many studies on ACP in patients with COPD, Houben et al showed in 2019 improved patient-physician communication about end-of-life care without causing psychosocial distress.^14^ In patients with lung cancer, ACP had significant positive effects on quality of life^15^ and, notably, a study in palliative care, including ACP, also significantly increased survival time.^16^ While the survival time has not been studied in patients with ILD, studies underpinned the importance of ACP and the positive impact on quality of life in interstitial lung diseases (ILD) patients.^17-19^

By explicating goals and preferences for future medical treatment and care, these are more likely to be fulfilled, even more when a personal representative is involved in this process.^12,13,20^ Patients felt their autonomy was supported and empowered by the informed health care decisions after having participated in one or more ACP conversations.^21^ Documentation of each ACP conversation with patient and relatives about their wishes and needs, supports continuity of care. ACP is recognized as an essential element of palliative care and is included in the national quality framework for palliative care.^22,23^

Although ACP is advised in the new guideline palliative care for COPD and ILD,^24^ ACP is not yet structurally implemented in the real life care for patients with PF in the Netherlands. Insight into current practices is lacking; this insight will add to the implementation of ACP in daily IPF care.

The aim of this retrospective audit was to gain insight into current ACP practices by HCP in IPF/PPF patient care in the Netherlands to optimize clinical practice and inform future research.

## Methods

### Design

A retrospective audit was performed on data from patients attending at a national referral center for PF in the Netherlands. Patients were eligible if IPF or PPF was diagnosed, and the patient had died between December 2017 and December 2020. Patients listed for lung transplant and patients without follow up in this hospital (seen only for second opinion) were excluded, see Flowchart 1. All patients had written biobank informed consent, and the study was approved by the Medical research Ethics Committees United.

**Flowchart 1.**
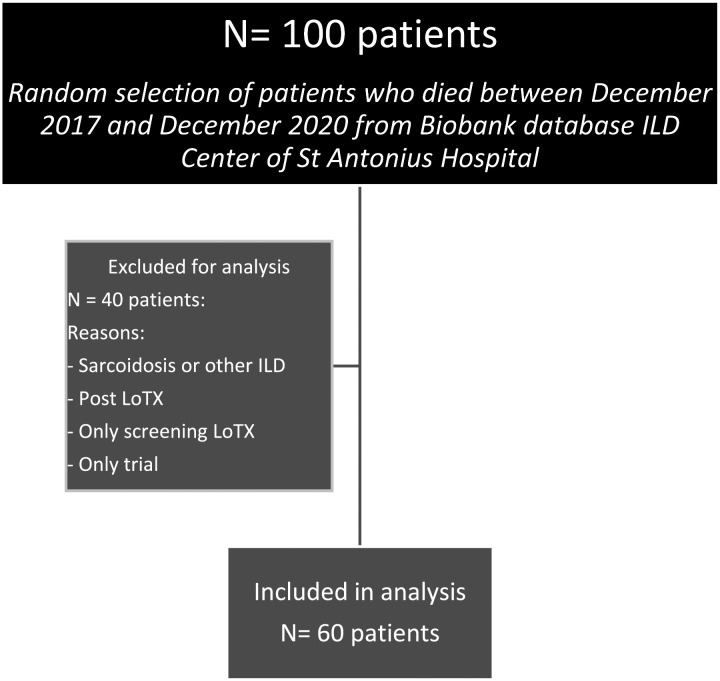
Patient selection.

### Data Collection

The primary outcome of this study was the frequency key elements were described in reports from conversations written by health care professionals (HCPs). For the data collection a data extraction model was developed (LT, MZ) based on the guideline for process and uniform documentation of ACP,^25^ the systematic review by Fahner,^10^ the ACP definition including recommendations by Rietjens et al^8^ and an example of a frequently studied and used ACP intervention Respecting Choices.^26^ Both LT and MZ identified concept in literature and discussed until consensus was reached. The ACP key elements were divided into two categories, (1) pragmatic elements, describing ACP elements on process and decision making and (2) emotional elements describing emotions related to end of life and dying. All ACP key elements are defined in Table 1.

**Table 1.** Data Extraction Model: Definition of ACP Key Elements and How They Were Collected From the Notes of the HCP.

Key Elements	
Pragmatic elements	Description
a. Goal of the conversation	Creating space to make plans for future care, or; the goal of an ACP conversation is explained to the patient
b. Personal representative	Personal representative; the person the patient has chosen to make decisions on their behalf if they are unable to speak or make decisions for themselves. This is dialogued and a name is noted in the chart
c. Knowledge of the illness	Patient shows knowledge about illness and (possible) changes in future health care status in relation to his disease
d. Knowledge of the complications	Patient shows knowledge about complications of the disease, even as preventive actions and risks Knowledge about resuscitation and the impact
e. Resuscitate order	Preference of the patient is dialogued and noted in the chart. This is not necessarily a non-treatment code, but can also be a full-treatment code, but rather that it has been dialogued and decided upon
f. Goals of treatment	Expectations and wishes about medical care are noted, eg,; selective treatment plus comfort focused care, only comfort focused care, participation in trials, strategy regarding hospitalizations
g. Goals of care	Expectations and wishes about care, eg,; optimizing quality of life, preferred place of last care, use of informal care, shared care
Emotional elements	
a. Vision on good life	Description by the patient about the meaning of ‘good life’ for him
b. Fears and concerns	Fears and concerns expressed by the patient or relative in relation to the disease of medical care
c. Hope	Hope expressed by the patient towards care and treatment. And the hope that follows when hope on within care and treatment is not realistic anymore
d. Experiences with deceased relatives	Experiences of the patient with relative(s) getting ill. Challenges and believes a patients has based in this experience
e. Religion	Religion and personal believes can influence the care or treatment of a patient especially in relation with care towards end of life
f. Euthanasia	Knowledge and wishes of the patient regarding euthanasia
g. Readiness	Patients’ readiness to participate in conversations about end of life. Aspects that show readiness is; starting the conversation them self, asking questions about their future
Process characteristics of the conversation	
Who initiated the conversation?	If clearly reported in the notes; who initiated the item dialogued in the conversation. If not clearly noted; the HCP is the assumed initiator
Who reported the conversation in de record?	The person who wrote and saved the note of the conversation
Location of the conversation	Location of conversation
	- Outpatient clinic
	- Inpatient clinic; pulmonology ward
	- At home/by telephone
	- Emergency department
Timing of the conversation	- Longer than 3 months before death
	- Between 1 and 3 months before death
	- Between 1 week and 1 month before death
	- Within 1 month before death
	- Bereavement conversation after death, with relatives
What was the occasion for this conversation?	- In case of acute progression
	- Case management (by nurse)
	- Regular consult
Who were involved in the conversation?	All participants of the conversation, eg,: patient, family, partner, pulmonologist, nurse, other disciplines (physiotherapist, GP, etc)
Which actions followed after the conversation?	a. Conversation treating pulmonologist
	b. Conversation other pulmonologist
	c. Conversation GP
	d. Conversation family (with or without HCP)
	e. Follow up consultation
	f. Conversation nurse
	g. Consultation of Palliative advisory board
	h. Consultation other discipline

Secondary outcomes were the process characteristics of the conversation: initiator of ACP, motive to start ACP, other attendees, location (outpatient clinic or inpatient clinic), and moment of the conversation (eg, 3 months before death).

Furthermore, patient and care characteristics were collected: age, diagnosis, pulmonary function tests, comorbidity, place of death and cause of death, number of hospitalizations in the last year and usage of shared care. The concept of ‘Shared care’ is an option for patients who have a long travel distance to our tertiary center to have alternate reviews in their local hospital.

### Data Analysis

A conceptual content analysis was performed.^27^ All notes from HCPs involved in the care for PF were reviewed, eg, pulmonologist, nurse, physiotherapist. Every note in the patients’ charts was coded with codes about timing, involved persons and location, combined with one of the 14 elements of ACP. Notes without elements of ACP were coded as ‘empty’ to gain insight into the number of conversations lacking elements of ACP. The minimum coding unit was one phrase. Coding was performed by 5 student nurses (AS, BD, LvdO, KW, PvB) and every first, second and tenth record was independently double coded by LT to ensure trustworthiness. Discrepancies were discussed in the research team (MZ, LT and nursing student) until consensus was reached. Afterwards, all records were checked by LT for completeness. Only coded data was used for the content analysis. Frequency of the elements, the content of the notes and the follow up data were analyzed. ATLAS.ti (v9) was used to manage, organize and code all data.^27^

Demographical information was characterized using descriptive statistics R studio version 4.1.2. Continuous data are summarized as mean with standard deviation or median with interquartile range (25%–75%) where appropriate. Categorical data are shown as number and proportion.

## Results

**Graph 1.**
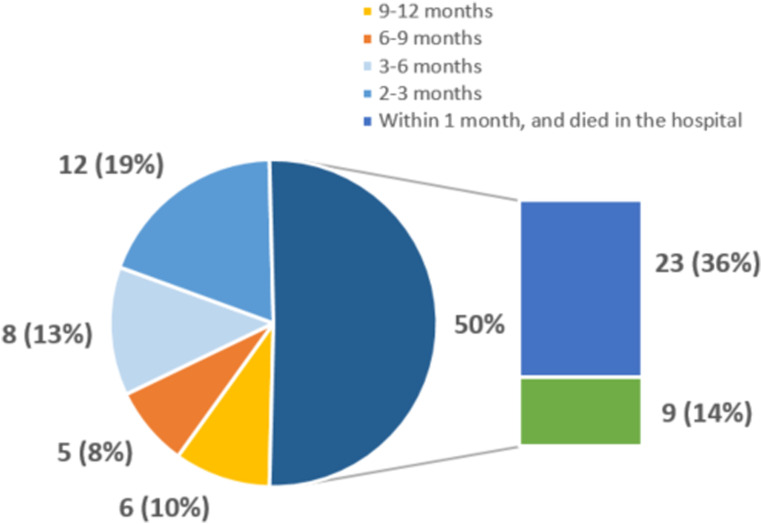
**Hospitalizations in last year of life**. Expressed in timeframes before death.

**Graph 2.**
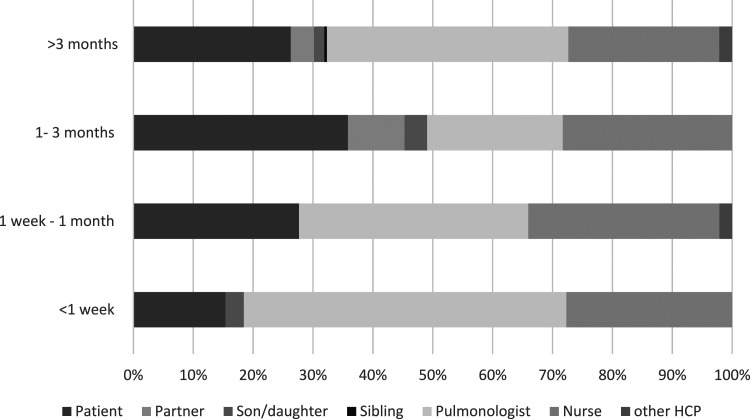
**Initiation of the conversation over time:** expressed in timeframes before death and shifting towards the end of life.

The charts of 60 patients are included: 45 (76%) male and mean age at death was 73 (7.7) (Table 2). Ninety-three% patients were diagnosed with IPF. The cause of death was respiratory failure for 65% of patients and for 15% of patients the cause of dead was not reported in the charts (see Graph 1 and 2).

**Table 2.** Demographics.

Characteristics	N = 60
Age at death	72.8 ± 7.7
Male	45 (76)
Diagnosis	
• IPF	56 (93)
• PPF (eg. fHP, NCIP)	4 (7)
Comorbidity	
• Pulmonary hypertension	15 (25)
• Heart failure	15 (25)
• Kidney failure	4 (7)
• Oncology	12 (20)
• Diabetes	8 (13)
Highest FVC^a^ predicted %	88.1 (73.1 - 98.9)
Highest DLCOc^b^ predicted %	46.4 (38.5-57.9)
Lowest FVC predicted %	62.6 (9.2-75.9)
Lowest DLCOc predicted %	28.5 (20.1-34.8)
Years of follow-up at ILD center	4 (2-5)
Shared care^c^	12 (20)
Oxygen therapy	44 (73)
Cause of death	
• Respiratory failure	39 (65)
• Cardio respiratory failure	2 (3)
• Medical condition unrelated to PF	5 (8)
• Euthanasia	5 (8)
• Unknown	9 (15)
Place of death	
• At home	19 (32)
• Nursing home	2 (3)
• Hospice	1 (2)
• Hospital	23 (38)
• Unknown	15 (25)

All variables in this table are in number (%), median (IQR) or mean ± standard deviation.

^a^FVC = forced vital capacity.

^b^DLCOc = corrected diffusing capacity for carbon monoxide, */**: Whilst serial FVC and DLCOc were collected, for practical reasons here only the highest and the lowest value is reported.

^c^The concept of ‘Shared care’ is an option for patients who have a long travel distance to our tertiary center to have alternate reviews in their local hospital.

In the last year of life, 35 patients (58 %) were hospitalized, with 63 hospitalization in total. Of these, 32(50%) occurred in the last months of life and 23 (37%) patients died in a hospital (see Graph 1).

In total, 2968 notes of conversations were coded of which 2025 (68%) hold no elements of ACP. Not one conversation was explicitly marked as an ACP conversation. In total, 919 (31%) conversations had at least one element of ACP and were included in the content analysis. There was no chart where every element of ACP (n = 0) was described. Notably, information on religion and the existence of personal representative were never noted (Table 3).

**Table 3.** Notes Found in the Charts About Specific Items. The Total Number in all Charts and One or More per Individual Patient Chart.

Pragmatic Elements (6)	Notes in Total (n = 919)	Notes in Individual Patient Charts (n = 60)
Knowledge of the illness	281	49 (82)
Knowledge of the complications	143	37 (62)
Personal representative^a^	0	0
Prognosis	39	14 (23)
Goals of treatment and care	691	51 (85)
Resuscitation order		25 (42)
Emotional elements (5)		
Experiences with dying relatives	20	8 (13)
Fears and concerns	236	49 (82)
Hope	54	18 (30)
Vision on good life/quality of life	99	27 (45)
Religion	0	0

All variables in this table are in number (%).

^a^Personal representative; the person the patient has chosen to make decisions on their behalf if they are unable to speak or make decisions for themselves.

### Pragmatic Elements

#### Knowledge of Illness and Complications

Knowledge of illness and complications were noted in many charts. Knowledge of illness and complications were often dialogued at the same time and therefore presented together. The knowledge of illness and knowledge of complications reported in the charts can be separated into 2 items; the knowledge/information which is transferred by the HCP to patient and relatives, and the knowledge the patients and relatives already have. In most charts it was not clearly noted which information was given by the HCP, what the patient understood, and which questions were asked by the patient. To illustrate, see quote 1 (Table 4).

**Table 4.** Quotes to Illustrate Results.

	Quote	Time Prior to Death	HCP/Contact Type
1	‘Information consult with patient and daughter; PowerPoint [about PF and medication] discussed; questions answered.’	>3 months	Nurse
2	‘[patient] Has a couple of questions. Answered these. It got much clearer for patient. He stated he had been given too much information to comprehend it all	>3 months	Nurse by telephone
3	‘Partner calls: patient has more stomach problems since Wed, when he raised the doses of pirfenidone to 3 × 3 tablets.’	>3 months	Nurse by telephone
4	“Talked about oxygen. Wants to try the Inogen [special oxygen system for mobility] first. Discussed that this might not be enough anymore shortly, because he has to control nose-breathing. [patient] Does not want cylinders yet.”	>3 months	Pulmonologist
5	‘[patient] Mentions he can do almost nothing anymore, but still enjoys the things he can.’	1- 3 months	Nurse by telephone
6	‘Nintedanib is the preferred treatment, because he [the patient] is often outside working [referring to skin burning from the sun].’	>3 months	Nurse
7	‘Patient concerns a lot about the further physical regression and the future.’	>3 months	Nurse
8	‘[Patient] Asks if there is a study in which he could participate.’	1 - 3 months	Pulmonologist
9	‘[Patient gets] Questions from his family if it is all worthwhile anymore. Patient wants to do anything that is still possible.’	>3 months	Pulmonologist
10	‘.. long conversation about the last phase of life. Patients has difficulties to talk about this, but understands it is necessary. Stated that she should think about her view on this.’	>3 months	Pulmonologist
11	‘Couple is trying to get a new apartment, …, [patient] wonders if he is going to survive long enough’	>3 months	Pulmonologist by telephone
12	‘Mother had PF, also treated here and died here. [patient] Is emotional about this and feels stressed what will follow towards the future for himself. (…) Does not like the oxygen. Has a traumatic experience regarding his mother.’	>3 months	Pulmonologist

Sometimes patients did not follow advices given by the HCP’s, HCP seemed to assume patients didn’t understand the information and information was clarified or repeated. For example, see quote 2.

There were consultations about symptoms and adverse effects of medication all initiated by patient or their relatives. To illustrate, see quote 3.

#### Goals of Medical Treatment and Care

The goals of the medical treatment and care were often described (691 notes (75%) in 51 charts), of which 71 (8%) short term goals and 52(6%) long term, or no specific timing. Long-term goals were often introduced by the HCP anticipating possible future problems, quote 4.

Medical treatment of PF with anti-fibrotic medication and the goals of the treatment were often described by the HCP. For example, slowing down the progression of the disease. In multiple charts it was written that the patient was not ready to start treatment with oxygen or morphine, and this always seemed to be dialogued with the patient.

Patients’ view on side effects of the medication before commencement were only described when it was a reason for a patient not to start or to postpone the use of a certain medication.

In the included charts it was seen that future care (eg, hospice care or care at home) was organized during hospitalization and described during hospitalization before discharge. Future care and care at home was almost never described in outpatient care. If a patient indicated to have palliative care needs or increased care needs, they were referred to their general practitioner.

#### Treatment Limiting Decisions

In 25 charts (42%), resuscitation and artificial respiration were mentioned. In charts where there was documentation about a conversation concerning resuscitation, some of these conversations were in the last month of life. In one case the treatment limiting decision was monitored and altered over time.

Options for symptom management were described in 26 patients’ charts (43%) mostly focused on breathlessness, the use of oxygen therapy and the use of morphine. Sometimes options to handle cough symptoms were described. Often a symptom was dialogued because the patient perceived a symptom that required an intervention. In a few notes this was an informative proactive conversation about future scenarios, eg, palliative care options for future symptoms.

#### Personal Representative

There was no documentation identifying a personal representative. In 6 charts it was clear a member of the family or the partner of the patient had an important role. In some notes this relative was contacted instead of the patient, but no written consent was present in the chart.

### Emotional Elements

#### Values in Life

In 27 charts, the patients’ values in life were described. Values, wishes and needs were mostly described using words as ‘quality of life’ or ‘comfort’. Most notes concerned the quality of life in the present, not towards their future, and often regarding possible side effects of medication, for example quote 5.

When patients are starting anti-fibrotic therapy, the argumentation for this choice was in some charts described, see quote 6.

#### Fears and Concerns

Fears and concerns of the patients and relatives were described in 48/50 charts and in total in 239 notes. Most described fears and concerns concerned the patient’s future, mostly related to the effect of the medication, but also about problems in the future. For example, quote 7.

In 4 charts, fear of choking was described. It was not described how much this influenced the patient’s life and which interventions were taken. When patients were admitted to the hospital, concerns in general were described more often and more in detail. There is no evidence found of a structural follow up after mentioning fears and concerns, no referrals to psychological care or speech therapist were mentioned.

#### Hope

Hope was mentioned 18 (2%) times, mostly contextual linked to hope for a positive effect of the therapy. To a lesser extent, hope related to a rapid discharge from hospital, study participation and having less hope on a successful treatment; described in quote 8 and 9.

#### Patients’ Readiness for ACP

In 50% of the charts signs of patients’ readiness for ACP were described; quote 10.

In some notes, HCPs noted questions or statements from patients about the future. These statements can be used to start a broader conversation about the future goals and preferences for the patient. However, in most of the notes it remained unclear if this statement really led to a conversation; quote 11.

#### Experiences with Deceased Relatives

In 8 charts there was documentation about deceased relatives. In most notes this were deceased first line relatives who died from PF as well. In a few notes the death of a partner (dying of another disease) was described. Mostly, the meaning of this loss and the patients’ coping was not described. Sometimes this experience was a reason for a specific decision. For example, quote 12.

#### Process Information

As shown in Table 5, most ACP topics were dialogued during a regular consultation (34%) or in case of acute progression (29%). In one-third of the conversations, the patient initiated a talk about ACP elements (36%). The other dialogues about ACP elements were initiated either by the pulmonologist (30%) or by the nurse (21%). These initiators seemed to change towards end of life; in these situations, the HCPs more often introduced an element of ACP (Graph 2).

**Table 5.** **Process Characteristics**. Characteristics of the Conversation in Which One or More Items of ACP was Noted in the Individual Patient Charts.

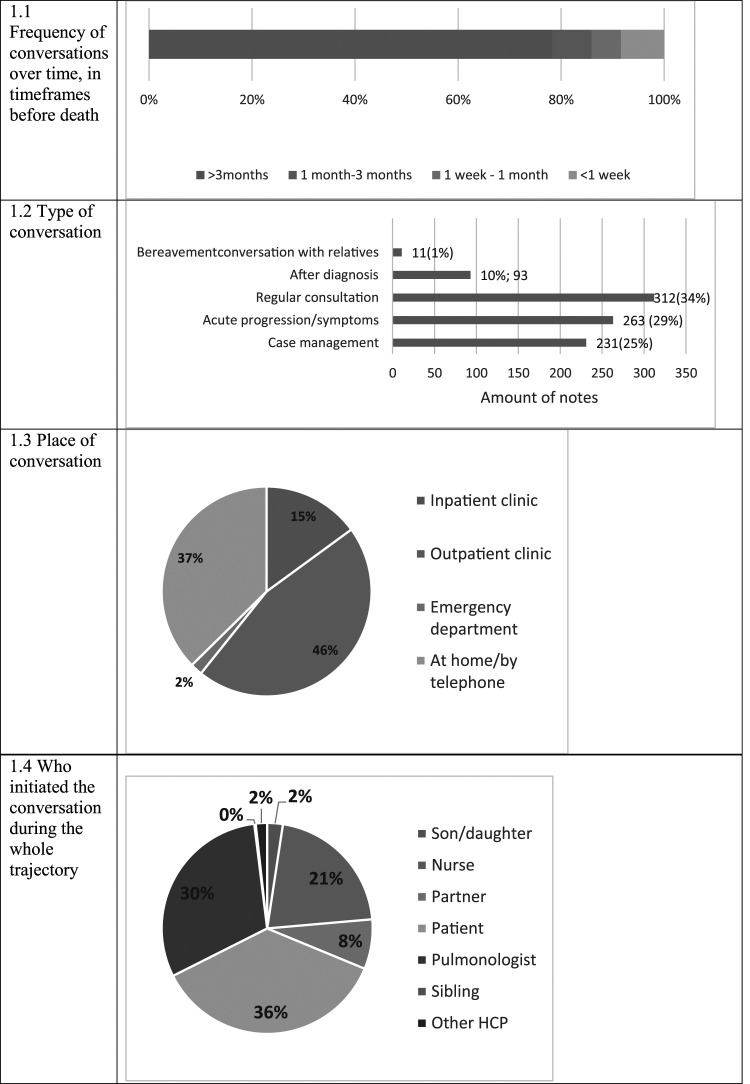

## Discussion

The aim of this retrospective audit was to gain insight into current ACP practices by HCP in IPF/PPF patient care in the Netherlands to optimize clinical practice and inform future research. We gained insight into pragmatic and emotional elements of ACP dialogued with patients in the current care for patients with PF. The study describes HCPs usual reporting in hospital charts of patients with PF regarding ACP, without the use of a reporting tool and without a standard protocol for ACP.

Results showed a wide range of ACP elements the HCP dialogued with patients and their relatives. Not 1 chart described all ACP elements. Pragmatic items of ACP such as goals of treatment and knowledge of the disease are more often described than the emotional items. These charts showed information was given, but the emotional reaction of the patient and/or his relatives to this information was absent in most notes. Documenting emotions in addition to the pragmatic items gives the HCP a better understanding of patients’ perspectives, supports continuity of care, and assists in planning appropriate future care.^28^

In the multidimensional approach of palliative care, one key aspect is the spiritual dimension. It appears that most HCPs don’t document about this dimension, for example concerning the philosophy of life. However, this may be crucial for the patient guiding their final goals and preferences.^29,30^

It is remarkable that resuscitation and intubation were noted only in 42% of the included charts. Literature showed intubation and ventilation in patients with IPF induced a high mortality.^11^ The pooled data in the study of Mallick et al showed an aggregated mortality of 87% among patients ventilated in intensive care units. Only patients with a post-operative respiratory failure showed a lower mortality after ventilation. These items should be dialogued with every patient and their relatives to prevent useless medical interventions.

Both examples might show the unfamiliarity of ACP and Shared Decision Making (SDM) within the ILD care. The steps of SDM (informing, explanation, dialogues about preferences, making the decision)^31^ might help to find questions to ask the patient regarding ACP and the emotional ACP elements and can be used in ACP conversations.

Our study showed a poor documentation of ACP elements in the regular hospital care for patients with PF without a documentation strategy or implementing ACP. These results are in line with a chart review in a geriatric primary care clinic.^32^ In this research only 34 of the 98 reviewed patient charts had documentation of ACP, also a similarly low prevalence of documentation. With patients getting older and more multi-morbid, and therefore need more physicians than ever before.^33,34^ The development of Electronic Health Record, wherein multiple disciplines read and document their work can play an important role in the documentation of elements and help to provide multidisciplinary appropriate care. A documentation strategy such as a standardized reporting form is available but is still unknown if this supports the HCPs documentation. In addition, implementation has to focus on follow up of the dialogues, since patients can alter their preferences during the illness trajectory.

To our knowledge, this is the first study that investigated the documentation of ACP elements in de care for patients with PF. A strength of this study is the reproducibility of the study, since ACP was defined and operationalized and elements of ACP were collected in a structured manner based on literature.

Some limitations must be considered. The data was collected for clinical purposes rather than research and the retrospective nature excluded active patient participation. The charts in this study may be incomplete due to the lack of guidelines, resulting in inconsistent use by different HCPs. Consequently, these results reflect only what has been reported, not what has actually been done or dialogued. Moreover, this paper does not capture the essentials of experienced communication from the patients perspective. A chart review only showed the perspective of the HCP. This retrospective study was cost effective and whilst a single center study in these rarer conditions is likely to translate well to other European clinical services.

## Conclusion

To conclude, this study in de IPF/PFF population revealed that the term ACP is not used in the charts, there are limited number of notes with elements of ACP and if there are notes, these are mostly pragmatic. Emotionally elements of ACP such as the reaction of the patient and their partners are even less frequent noted.

Future (implementation) research should focus on implementing palliative care and multidimensional symptom management and related documentation strategy. ACP can be a helpful intervention to get more attention and insight into the emotional elements. Comprehensive documentation of these dialogues are key for continuity of care.
